# Identify changes of brain regional homogeneity in early and later adult onset patients with first-episode depression using resting-state fMRI

**DOI:** 10.1371/journal.pone.0184712

**Published:** 2017-09-14

**Authors:** Zonglin Shen, Linling Jiang, Shuran Yang, Jing Ye, Nan Dai, Xiaoyan Liu, Na Li, Jin Lu, Fang Liu, Yi Lu, Xuejin Sun, Yuqi Cheng, Xiufeng Xu

**Affiliations:** 1 Department of Psychiatry, The First Affiliated Hospital of Kunming Medical University, Kunming, Yunnan, China; 2 Department of Medical Imaging, The First Affiliated Hospital of Kunming Medical University, Kunming, Yunnan, China; 3 Mental Health Institute of Yunnan Province, Kunming, Yunnan, China; Institute of Psychology, Chinese Academy of Sciences, CHINA

## Abstract

**Objective:**

Previous work exhibited different brain grey matter volume (GMV) changes between patients with early adult onset depression (EOD, age 18–29) and later adult onset depression (LOD, age 30–44) by using 30-year-old as the cut-off age. To identify whether regional homogeneity (ReHo) changes are also different between EOD and LOD by using same cut-off age, we used resting-state functional magnetic resonance imaging (fMRI) to detect the abnormal ReHo between patients with EOD and LOD in the present study.

**Methods:**

Resting-state fMRI scans of 58 patients with EOD, 62 patients with LOD, 60 young healthy controls (HC), and 52 old HC were obtained. The ReHo approach was used to analyze the images.

**Results:**

The ANOVA analysis revealed that the ReHo values in the frontoparietal, occipital, and cerebellar regions were significantly different among the four groups. Relative to patients with LOD, patients with EOD displayed significantly increased ReHo in the left precuneus, and decreased ReHo in the right fusiform. The ReHo values in the left precuneus and the right fusiform had no significant correlation with the score of the depression rating scale or illness duration in both patient subgroups. Compared to young HC, patients with EOD showed significantly increased ReHo in the right frontoparietal regions and the right calcarine. Furthermore, the increased ReHo in the right frontoparietal regions, right insula and left hippocampus, and decreased ReHo in the left inferior occipital gyrus, right middle occipital gyrus, left calcarine, and left supplementary motor area were observed in patients with LOD when compared to old HC.

**Conclusions:**

The ReHo of brain areas that were related to mood regulation was changed in the first-episode, drug-naive adult patients with MDD. Adult patients with EOD and LOD exhibited different ReHo abnormalities relative to each age-matched comparison group, suggesting that depressed adult patients with different age-onset might have different pathological mechanism.

## Introduction

Major depressive disorder (MDD) is an affective disorder with clinically heterogeneous features. Previous study found depressed patients with different age of onset have different clinical features[1–4]. For examples, relative to later onset (LO) depression, patients with early onset (EO) depression are usually with higher positive family history[1], higher suicidal thought[3]or suicide attempts[4], and commonly comorbid with personality disorder[5]. Some other previous studies also found the EO depression was associated with more anxiety than LO depression[6, 7]. A meta-review also indicated that the different age-onset depression might be different subtypes of depression[8]. In addition, the heritability of LO depression (>30 years) is lower than that in EO depression (<30 years) (10% vs 47% respectively)[9]. In summary, these clinical findings together with the diverse heritability implicated that depression with different age of onset might have different pathological mechanism.

However, the clinical features mentioned above between EO and LO depression were depended on variety cut-off age, there is inconsistency with the cut-off age for early- and later-onset depression. A previous study considered the EO depression as the first episode occurring before age 18 years[10], whereas, another study also considered the EO depression as the first episode occurring before age 30 years[2, 5]. On the other hand, the LO depression was defined as the first episode occurring after age 40[3] or 50[2] or 60[4] years. The biological evidence for the cut-off age in these studies remains unclear. Thus far, there is no consensus upon the age of onset in defining EO vs. LO MDD.

For decades, the neuroimaging has been widely used to explore the pathomechanism of MDD. The regional homogeneity (ReHo) was used to detect a given voxel that is temporally similar to its neighbors within a single region[11]. The abnormal ReHo reflects the changes of temporal aspects of neural activity in the regional areas. Additionally, the ReHo method has been used to investigate the neural activity change in depression[12–14] and a ReHo study provided some evidence to distinguish EO and LO MDD[15]. To our knowledge, there was only one study used ReHo to analyze the resting-state functional magnetic resonance imaging (fMRI) data to distinguish patients with EO MDD from those with LO MDD[15]. The study revealed that patients with EO MDD and those with LO MDD have abnormal neural activities in different brain regions. However, there was a large age gap between EO MDD and LO MDD in their study. The age of patients with EO MDD were under 35 years while the age of patients with LO MDD were over 60 years in their study[15]. Thus, the actual abnormal neural activities between EO MDD and LO MDD remains unclear. On the other hand, the onset age after 45 years was associated with increased familial risk of vascular disease in MDD[16], suggesting the pathological mechanism of MDD that occurs after age 45 years might be different from MDD occurs before the age of 45 years. What is more, one recent study from ENIGMA found the smaller hippocampus volume in MDD might moderated by age of onset[17], suggested the age of onset is a great important factors in analyzing anatomical change in MDD patients and highlighting the age of onset should be considered carefully in detecting pathomechanism of MDD. More recently, one of our studies found that early adult onset depression (EOD) and later adult onset depression (LOD) exhibited different whole brain grey matter volume (GMV) by using 30-year-old as the cut-off age[18]. Moreover, our another study also found specific abnormalities in the brain circuitry between EOD and LOD by using the same cut-off age[19]. So it seems 30 years might be an appropriate cut-off age for distinguishing EOD and LOD. However, previous studies found there was a dissociation pattern of brain anatomical deficits and functional change[20, 21], especially with regard to GMV and ReHo[20], which suggested brain functional and anatomical change might contributed independently to pathomechanism of MDD.

Therefore, based on our previous work and avoiding the vascular disease in older patients (>45 years old), we sought to examine whether the MDD patients with the onset age between 18–44 years old (the age was 18–45 years) could also be distinguished by abnormal ReHo in the present study. We hypothesized that the EOD (age 18–29) and LOD (age 30–44) patients would have different regional neural activities.

## Materials and methods

### 1.1 Ethics statement

All participants and guardians gave written informed consent. This study was approved by the Ethics Review Board of Kunming Medical University, Kunming, Yunnan Province, P. R. China.

### 1.2 Participants

All patients were recruited from the outpatient clinic or inpatient wards of the Psychiatry Department of the First Affiliated Hospital of Kunming Medical University. Right-handed, 147 MDD patients (50 males and 97 females) met the criteria were recruited in the depression group. The inclusion criteria were as follows: ① The diagnosis of MDD was independently made by two experienced psychiatrists in accordance with the Diagnostic and Statistical Manual of Mental Disorders, fourth edition (DSM-IV, American Psychiatry Association, 1994), ② first episode without a history of antidepressants treatment, ③ be aged between 18–45 years, ④ the total score of 17-item Hamilton Depression Rating Scale (HDRS) was not less than 17, ⑤ right handedness, ⑥ the patients or their legal guardian signed the informed consent form. The exclusion criteria included the following items: ① having a history of Axis I psychiatric disorders. ② having a history of neurological illnesses or other severe diseases, ③ having a history of brain injury or obvious psychiatric symptoms, ④ with substance abuse, ⑤ having received electroconvulsive therapy (ECT), transcranial magnetic stimulation(TMS) or systematical psychotherapy, ⑥ pregnant or nursing women and ⑦ having physical limitations prohibiting them undergoing MRI scans.

130 sex-, and age-, matched healthy controls (49 males and 81 females) were also recruited in the healthy control group and excluded: ① with a family history of psychiatry, ② with a psychiatry disorder, ③ with a severe physical disease and/or neurological disease, ④ with substance abuse, ⑤ with a history of brain injury, ⑥ pregnant or nursing women, or ⑦ inability to undergo a MRI scan.

All participants underwent the MRI scans. All of the participants were recruited from February 2012 to July 2015. Each MDD patient and HC was assigned a number at the time of recruitment. Their data including demographic information, clinical and imaging data can be indentified through the number but not their name. The checklist is available as supporting information (see S1 Checklist)

### 1.3 Methods

#### 1.3.1 Clinical materials and subgroups

Data were collected from all participants including age, sex, education level and the duration of depression. The depressive symptoms were assessed by an experienced psychiatrist using the 17-item Hamilton Depression Rating Scale (HDRS, Hamilton 1960). Based on previous research[18, 19], all patients were divided into two subgroups: the EOD group (18–29 years) and the LOD group (30–44 years). The HC were correspondingly divided into two subgroups: the young HC and the old HC.

#### 1.3.2 Image acquisition

MRI scanning was performed by a skilled radiological technician on a Philips Achieva 3.0-T MRI scanner. Restraining foam pads were used to minimize head motion. Participants were also informed to keep eyes closed and without thinking during scanning. Normal T1 and T2-weighted MRI scans were first performed to exclude obvious structural abnormalities. The parameters were as follows: time of repetition (TR)/time of echoing (TE) = 2500/80ms, slice thickness = 6 mm, field of vision (FOV) = AP(250mm)×RL(193mm)×FH(142mm), matrix size = 128×128, flip angle = 90°, slices = 16, gap = 2mm, scan duration time = 45sec.

The resting-state functional images were acquired by using an echo-planar imaging sequence with the following parameters: TR/TE = 2200/35ms, flip angle = 90°, FOV = 230×230mm, matrix = 128×128, slice thickness = 3.0mm without interlayer spacing, slices = 50, scan duration time = 17min40sec. All of the sections were acquired parallel to the anterior-posterior commissure line.

### 1.4 fMRI imaging preprocessing

All primary DICOM images were converted into NIfTI format using the MRIConvert software (http://lcni.uoregon.edu/downloads/mriconvert/mriconvert-and-mcverter). Then, fMRI image preprocessing was performed in the statistic parametric mapping software package (SPM8,http://www.fil.ion.ucl.ac.uk/spm) running in the Matlab 2012a (MathWorks, Natick, MA, USA) and in the Data Processing Assistant for Resting-State fMRI (DPARSF, http://rfmri.org/DPARSF) [22]. The steps of preprocessing were as follows: First, the first 10 volumes were discarded and the slice timing and head motion were corrected. Second, the spatial normalization was performed by using the standard EPI template in the Montreal Neurological Institute (MNI) space in SPM8 and then normalized images were resampled to 3×3×3 mm^3^. Finally, the resulting data were filtered at 0.01–0.08Hz frequency range to reduce low-frequency drift and high-frequency physiological respiratory and cardiac noise.

### 1.5 ReHo calculation

The ReHo calculations were also performed automatically by using the DPARSF software. Briefly, Individual ReHo mappings were generated by calculating the Kendall's coefficient concordance (KCC) of the time series of a given voxel with those of its nearest neighbors[23]. The number of neighboring voxels was set as 26. The consistency and similarity for each individual were assessed by calculating the KCC of the time series of one given voxel with those of its adjacent voxels in a voxel-wise analysis assuming that a voxel was temporally similar to those of its neighbors[24]. To reduce the influence of individual variations, the normalization of ReHo mapping was carried out by dividing the KCC among each voxel by the average KCC of the whole brain for each subject[25, 26]. Finally, the resulting mapping was spatially smoothed by convolution with a 4-mm full-width at half-maximum (FWHM) Gaussian kernel.

### 1.6 Statistical analysis

Statistical analyses were performed using the Statistical Package for the Social Sciences (SPSS 17.0 for Windows). The distributions of age and years of education of the four groups were compared by using one-way analysis of variance (ANOVA), and the chi-square test was used to compare the sex distribution. Two sample *t*-tests were used to compare the illness duration and HDRS score between the two patient subgroups. The significance threshold was set at p<0.05.

A voxel-based ANOVA comparison of the whole brain ReHo maps among the four groups was performed in REST software (http://restfmri.net/forum/REST_V1.8). The statistical threshold was set at p<0.05 after false discovery rate (FDR) correction with an extent cluster of 100 contiguous voxels or greater, by using age, sex, grey matter volume and education level as covariates. Then post hoc t-tests were conducted to identify voxel-wise difference on the ReHo maps between each pair of groups by using the same covariates mentioned above (p<0.05, corrected for FDR with an extent of more than 100 contiguous voxels). In addition, both EOD and LOD patients were drug-naive and in their first episode, with a short illness duration. In order to detect subtle abnormal regional neural activities between the two subgroups, a more lenient statistical threshold (p<0.05 uncorrected and an extent threshold of more than 100 voxels) was applied when no significant result was found at p<0.05 FDR corrected.

To investigate the relationship between abnormal regional neural activities and clinical features, brain regions with abnormal ReHo were identified as regions of interest. Mean values of ReHo were extracted using the REX toolbox (http://gablab.mit.edu/downloads/rex.m). Pearson partial correlations were conducted between these abnormal values of ReHo and HDRS score or illness duration in each patient's subgroup in SPSS, controlling for age, sex, and education level in SPSS. The threshold of p<0.05 was considered to be significant for these analyses.

## Results

### 2.1 Demographic data

In order to detect actual differences among different subgroup, 45 subjects (14 EOD, 13 LOD, 12 Yong HC, and 6 Old HC) were excluded due to excessive head movement (>1.5°and > 1.5 mm). Finally, 58 patients with EOD, 62 patients with LOD, 60 young HC, and 52 old HC were included in this study. The demographic information and clinical features were presented in Table 1. There was no significant difference in age or sex between MDD and HC, as well as between EOD and LOD relative to each age-matched comparison group. However, since the control subjects had a higher educational level than depressed patients, years of education was used as a covariate in the analysis. There was no significant difference in terms of illness duration, HDRS score between the two patient groups.

**Table 1:** The demographics and clinical characteristics of the sample.

**Variables** **(mean±SD)**	**HC**		**MDD**		**t(F)/χ2**	**p-value**
Sex(male/female)	41/71		42/78		0.065	0.891 ^a^
Age (years)	30.29±6.99		30.58±7.41		0.296	0.768 ^b^
Years of education	15.42±3.82		12.18±4.20		-6.139	0.000 ^b^
HDRS score	0.47±0.69		23.47±4.54		53.064	0.000 ^b^
GMV(mm^3^)	619.11±60.64		608.68±55.99		-1.363	0.580
	**Young HC**	**Old HC**	**EOD**	**LOD**		
Sex(male/female)	25/35	16/36	27/31	15/47	8.025	0.046 ^c^
Age (years)	24.63±2.37	36.83±4.33	23.98±2.89	36.74±4.38	\	0.000 ^d^
Age onset (years)	\	\	23.23±2.97	36.06±4.53	\	0.000 ^b^
Illness duration(months)	\	\	8.98±7.06	8.24±7.89	0.539	0.591 ^b^
Years of education	16.57±2.34	14.09±4.71	14.00±3.41	10.47±4.17	27.586	0.000 ^e^
HDRS score	0.42±0.65	0.54±0.75	22.91±4.19	23.98±4.82	-1.295	0.198 ^f^
GMV(mm^3^)	637.27±60.14	598.16±54.64	629.71±51.61	589.01±53.05	10.870	0.000 ^g^

MDD = major depressive disorder; EOD = early adult onset depression; LOD = later adult onset depression; HC = healthy control; HDRS = Hamilton Rating Scale for Depression.

^a^ The p-value for sex distribution in the two groups was obtained by chi-square test.

^b^ The p-values were obtained by two sample *t*-test.

^c^ The p-values for sex distribution among the four groups was obtained by chi-square test p = 0.711(Young HC vs EOD), p = 0.432(Old HC vs LOD).

^d^ The p-values were obtained by one-way analysis of variance tests. Post hoc *t*-test: p = 0.184(EOD vs Young HC), p = 0.918(LOD vs Older HC).

^e^ The p-values were obtained by one-way analysis of variance tests. Post hoc *t*-test: p = 0.000(EOD vs Young HC), p = 0.000(LOD vs Older HC), p = 0.000(EOD vs LOD).

^f^ The p-values were obtained by two sample *t*-test.

^g^ The p-values were obtained by one-way analysis of variance tests. Post hoc *t*-test: p = 0.342(EOD vs Young HC), p = 0.705 (LOD vs Older HC).

### 2.2 Abnormal ReHo among groups

**Fig 1:**
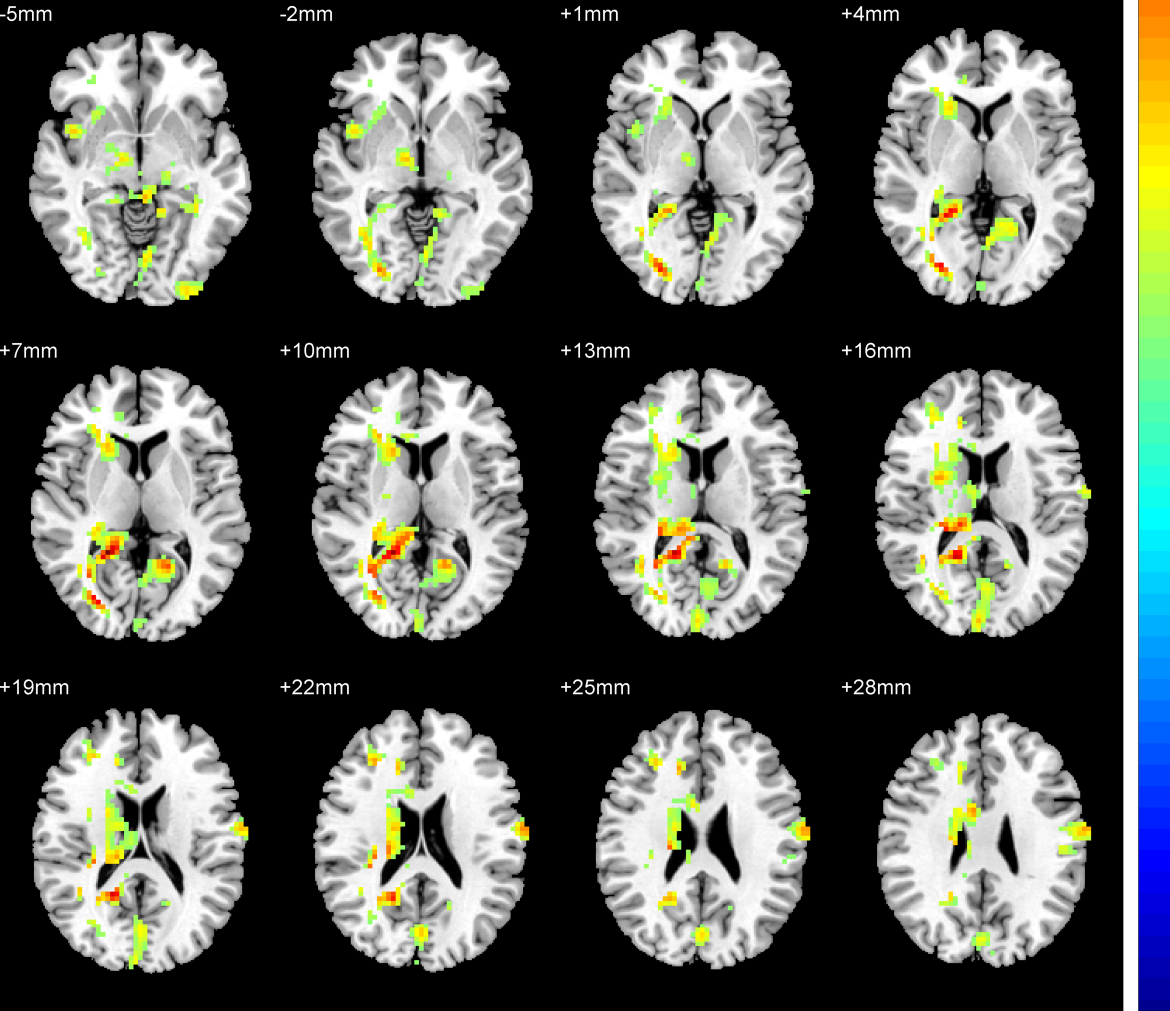
Statistical maps showing ANOVA result of ReHo abnormalities among patients with EOD or LOD and healthy control (FDR corrected). Red color: significant increased ReHo.

Fig 1 showed the one-way ANOVA analysis of the ReHo value among the four groups with age, sex, grey matter volume, and education level as covariates. Significant group differences of ReHo were found in the left postcentral gyrus, left supplementary motor area, left inferior occipital gyrus, left calcarine, left cerebellar posterior Lobe, left cerebellar_7b, and right calcarine. (Table 2, Fig 1).

**Table 2:** Regions of ReHo deficits in patients with MDD or EOD or LOD compared with HC.

Brain region	Side	Cluster size(Voxels)	MNI Coordinates (mm)			Z-Score	*p* value
			x	y	z		
**EOD<young HC**							
NA							
**EOD>young HC**							
Middle frontal gyrus extend to postcentral gyrus	R	262	27	-39	39	5.0518	<0.05
Calcarine	R	532	21	-48	6	5.479	<0.05
**LOD<old HC**							
Inferior occipital gyrus	L	263	-30	-96	-6	-4.95	<0.05
Calcarine	L	594	-15	-54	6	-5.5784	<0.05
Middle occipital gyrus	R	100	51	-78	6	-4.7043	<0.05
Supplementary motor area	L	381	-9	0	78	-5.4324	<0.05
**LOD>old HC**							
Middle frontal gyrus extend to postcentral gyrus	R	1063	39	-27	36	5.1626	<0.05
Hippocampus	L	127	-30	-30	-6	4.7932	<0.05
Insula	R	213	45	6	-3	4.3938	<0.05
**EOD<LOD**							
Fusiform	R	121	45	-21	-18	-3.4599	<0.05*
**EOD>LOD**							
Precuneus	L	318	-12	-66	60	3.3342	<0.05*

x,y,z are the coordinates of primary peak locations in the MNI space; Z is the statistical value of peak voxel showing ReHo differences among the EOD, LOD subgroups and healthy subjects. P<0.05, FDR corrected, cluster size≥100

* P<0.05, uncorrected, cluster size≥100.

EOD = early adult onset depression; LOD = later adult onset depression; HC = healthy controls.

### 2.3 Abnormal ReHo in EOD patients versus young HC

**Fig 2:**
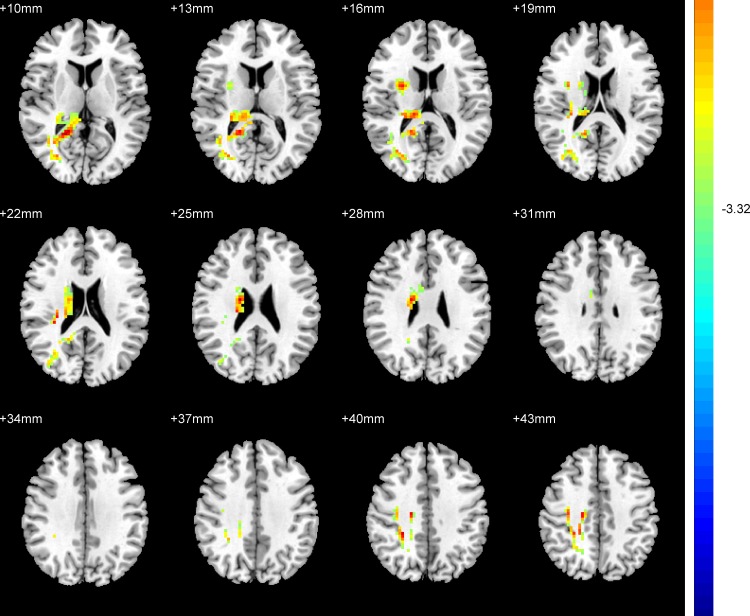
Brain regions showing the ReHo abnormalities in patients with EOD compared with young HC (FDR corrected). Red color: significant increased ReHo.

Compared with young HC, patients with EOD showed significantly increased ReHo in the right frontoparietal regions and the right calcarine. (Table 2, Fig 2).

### 2.4 Abnormal ReHo in LOD patients versus old HC

**Fig 3:**
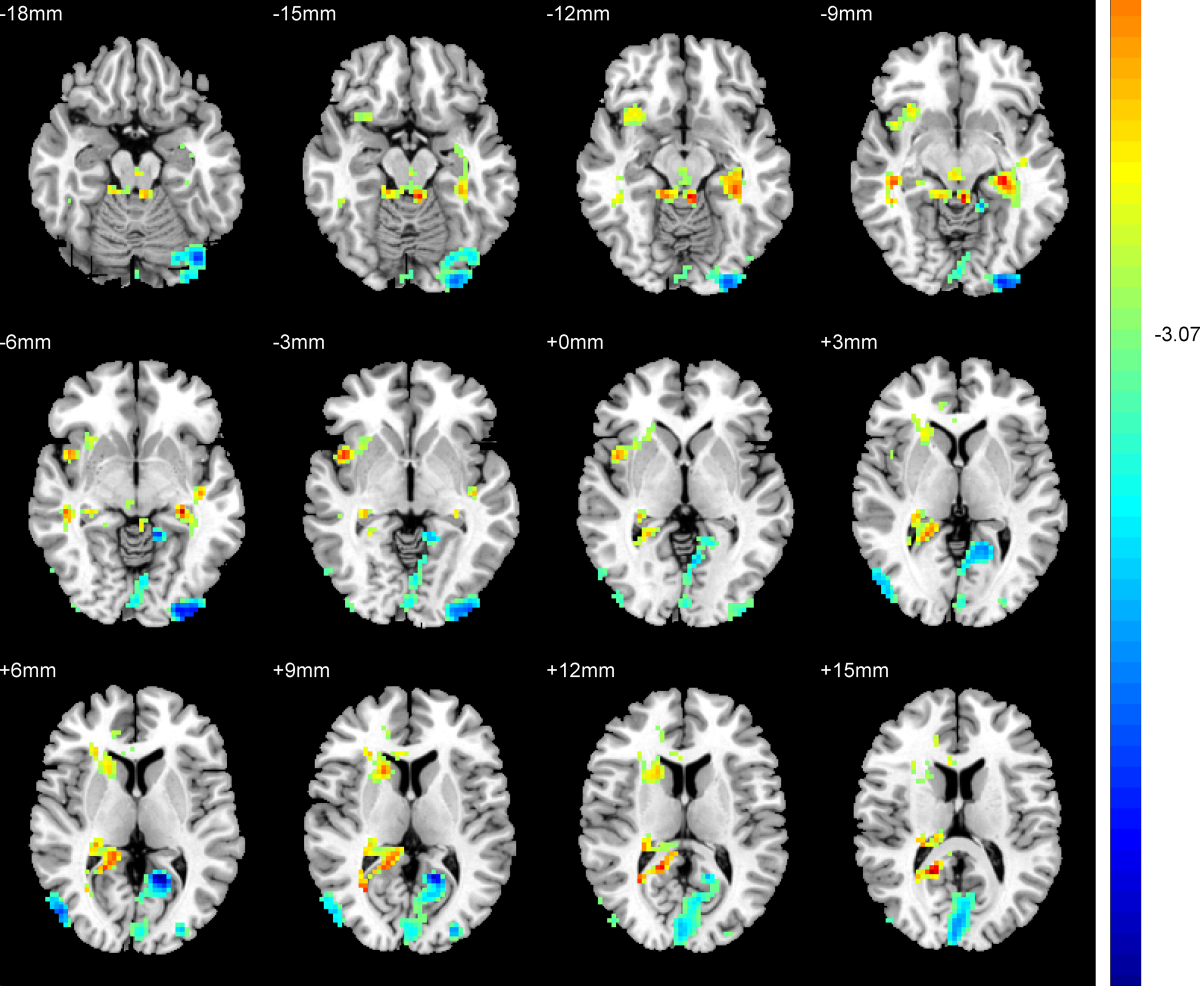
Brain regions showing the ReHo abnormalities in patients with LOD compared with old HC (FDR corrected). Red color: significant increased ReHo.

Compared with old HC, patients with LOD exhibited significantly increased ReHo in the right frontoparietal regions, right insula, and left hippocampus, and decreased ReHo in the right middle occipital gyrus, left inferior occipital gyrus, left calcarine, and left supplementary motor area (Table 2, Fig 3).

### 2.5 Abnormal ReHo in patients with EOD versus patients with LOD

**Fig 4:**
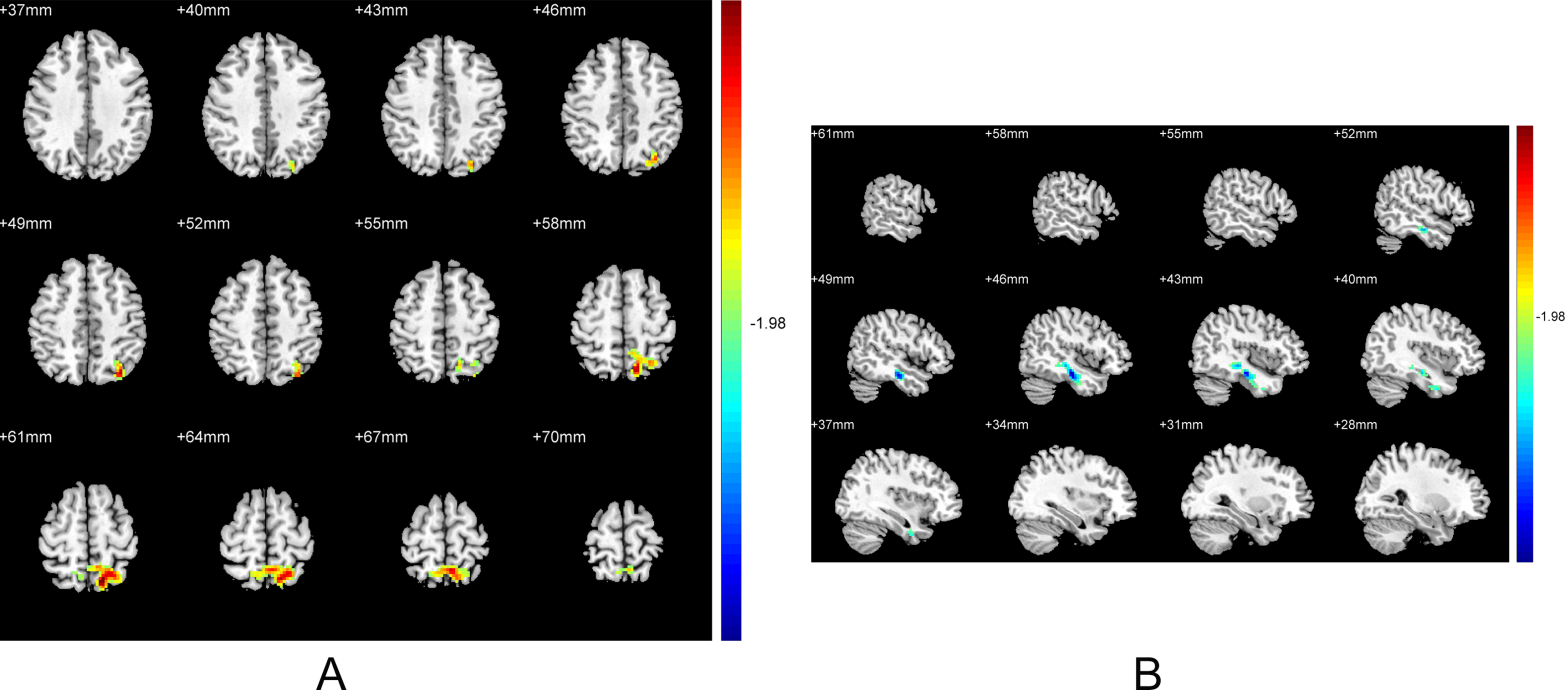
Brain regions showing the ReHo abnormalities in patients with EOD compared with LOD. **(A)** Relative to patients with LOD, the ReHo increased in left precuneus in patients with EOD (p<0.05, uncorrected). **(B)** Relative to patients with LOD, the ReHo decreased in right fusiform in patients with EOD (p<0.05, uncorrected). Red color: significant increased ReHo. Blue color: significant decreased ReHo

Relative to patients with LOD, patients with EOD displayed significantly increased ReHo in the left precuneus (Table 2, Fig 4 A), and decreased ReHo in the right fusiform (Table 2, Fig 4B).

### 2.6 The correlation of the ReHo and HDRS score or illness duration

Two brain regions (the right fusiform and the left precuneus) demonstrated ReHo difference between EOD and LOD. We used the REX toolbox to extract the mean values of ReHo in the right fusiform and the left precuneus in both patient groups respectively. There was no significant correlation between the ReHo value of the right fusiform and left precuneus and the symptom severity (HDRS) in neither the EOD group (r = -0.080 p = 0.562; r = 0.074 p = 0.590) nor the LOD group (r = 0.151 p = 0.254; r = -0.048 p = 0.719). Similarly, no significant correlation was observed between the ReHo value of the two brain regions and illness duration in neither the EOD group (r = 0.095 p = 0.488; r = 0.005 p = 0.970) nor the LOD group (r = -0.023 p = 0.865; r = 0.094 p = 0.478) after controlling for sex, age and education level.

## Discussion

In our previous study, we found specific regional GMV differences between patients with EOD and LOD. For further clarifying the core neuropathology of MDD, we used the approach of regional homogeneity to explore the regional neural activities in the same large sample in the present study. Firstly, widespread abnormalities of ReHo throughout frontoparietal, limbic regions, occipital cortex, and cerebellum were observed in patients with MDD. The findings are partly consistent with previous ReHo studies[27, 28]. These brain regions are important components of the limbic-cortical-striatal-pallidal-thalamic (LCSPT) circuit[29], which suggests that the pathogenesis of MDD is related to the abnormal function of the LCSPT circuit. Secondly, the most important finding was that patients with EOD or LOD exhibited different ReHo abnormalities when compared to each matched HC subgroup. A different form of ReHo abnormalities was also observed when compared the EOD subgroup to the LOD subgroup.

In the present study, relative to LOD, patients with EOD exhibited ReHo increased in the left precuneus and decreased ReHo in right fusiform. The ReHo increased in precuneus between EOD and LOD was in line with the finding of a previous study which revealed the EO MDD group had significantly increased ReHo in precuneus compared to LO MDD group[15]. They speculated the ReHo value increased in precuneus might be a diagnostic marker to distinguish EO MDD from LO MDD. Moreover, the bilateral precuneus (PCu)/posterior cingulate cortex (PCC) is the core region of the default mode network (DMN) at resting-state[30, 31]. Previous neuroimaging studies have exhibited that the MDD patients displayed altered activity patterns in the DMN [31, 32]. Abnormal functional of DMN might be characteristic of first-episode, drug-naive patients with MDD was supported by a Meta review[33]. On the other side, the genetic factors that influence DMN connectivity, the heritability for DMN was 0.424±0.17[34]. Further, the evidence of functional connectivity of DMN was different between the adolescents who were at high familial risk for developing clinical depression and those who were at low familial risk for depression was also found in previous study[35], supporting the DMN connectivity may also be influenced by genetic factors in patients with MDD. Considered the heritability[9] and family history[1] of EOD is higher than that in LOD, it is speculated the ReHo value deficit in precuneus within DMN between EOD and LOD patients might be caused by genetic factors. In addition, the DMN has been showed to be involved in self-referential activities[36] and related to negative rumination in depression[37]. The precuneus also related to consciousness, such as self-reflection, self-awareness and episodic memory[38]. On the other side, relative to healthy control, decreased regional homogeneity in the right precuneus in late life depression was also reported in previous study[27], suggesting the important role of PCu in the pathophysiology of the late stage of depression. Thus, the decreased ReHo in the precuneus of patients with LOD might implicate that the consciousness deficit associated with self-reflection, self-awareness and episodic memory is more severe in patients with LOD than those with EOD. Moreover, the GMV decrease in the right PCC/ PCu was observed in patients with EOD compared to those with LOD in our previous study[18], which further suggested PCC/ PCu is a potential region of distinguishing EOD from LOD. In addition, no significant correlation between HDRS score or illness duration and the left precuneus ReHo may imply that ReHo increased in the left precuneus was independent of depressive symptom severity or illness duration. Therefore, these findings suggested that the left precuneus may be an appropriate brain region for differentiating patients with EOD and LOD at the neural activity level.

The fusiform gyrus, another finding between patients with EOD and patients with LOD, is a part of visual cortex. The visual cortex was reported to have abnormal neural activities in patients with MDD[39]. The amplitude of low frequency fluctuation (ALFF) decreased in the right fusiform gyrus has been observed in first episode, treatment-naive patients with MDD[40]. A previous study suggested the fusiform together with temporal gyrus, posterior cingulate cortex, right angular gyrus and right parahippocampus gyrus may be related to cognitive processing[41]. Another study suggested the fusiform, inferior occipital gyrus and superior temporal gyrus composed the key regions of processing emotional facial expressions[42]. In addition, biases in interpretation of emotional facial information and in processing affective stimuli may be one of underlying mechanisms in the development of MDD in young people[43–45], and has been proved related to dysfunction of fusiform gyrus[44]. Furthermore, Our previous work found that relative to young healthy controls, patients with EOD showed GMV reduction in the right fusiform [18], indicating patients with EOD might have deficits in processing emotional facial stimuli. The ReHo value decrease in the fusiform was observed in patients with EOD compared to those with LOD in the present study, which implicated that the deficit in emotional facial stimuli which reflected the cognitive processing might be more severe in patients with EOD than patients with LOD. What is more, an MRI twin study revealed that the GMV reduction in fusiform gyrus were related to a genetic risk for depression[46], which highlighted the fusiform gyrus would be an important brain region related to genetic factors in depression. Additionally, the heritability of early onset depression is higher than that in late onset depression[9]. Thus, it is speculated that the ReHo value alteration in fusiform gyrus might be caused by genetic factors which reflected the heritability is different between patients with EOD and LOD. Nevertheless, the ReHo value of right fusiform was not correlated with HDRS score or illness duration, which also indicated the right fusiform might be another potential region for distinguishing EOD and LOD at the neural activity level.

LOD Patients showed an increased ReHo value in the hippocampus and insular. A wealth of studies on patients with MDD revealed that the hippocampus plays a crucial role in the pathologic mechanism of MDD[47]. Evidence from the Enhancing Neuro Imaging Genetics Through Meta Analysis (ENIGMA) study[17] and our previous study[18] revealed that the hippocampus volume in MDD might be moderated by age of onset. Our previous study also suggested that the pathological mechanism of LOD might be related to a smaller hippocampus volume. However, previous studies suggested the brain anatomical and functional abnormalities might independently contribute to the pathomechanism of MDD[20, 21]. Thus, the abnormal ReHo value in the left hippocampus might also be related to the pathogenesis of LOD MDD. The increased ReHo value in the left hippocampus in the present study may be a functional compensation for the morphological changes of the right hippocampus in LOD patients. The insular cortex connects with fronto-limbic regions[48]. Previous studies found that the metabolism in insular cortex would enhance when recalling sad events in healthy subjects[49]. The degree of metabolism enhancement in insular was associated with irritability[50] and sleep disorders [51] in MDD patients. Since different age-onset patients have different symptomatology[52], the increased ReHo value of the right insular might be one of the underlying neural patterns for the clinical symptoms of LOD.

We also observed an abnormal ReHo value in the left supplementary motor area (SMA), left inferior occipital gyrus (IOG) and right middle occipital gyrus (MOG) in LOD patients. The decreased ReHo value of the SMA in LOD is consistent with a previous study[27] that reported an increased ReHo value of the bilateral SMA in first-episode treatment-naive patients with late life depression (LLD). The occipital gyrus are components of the visual areas. Previous meta-analysis reported that the first-episode, drug-naive major depressive disorder patients showed decreased brain activity in numerous brain regions including the visual areas within the occipital lobe[33]. In addition, previous study also revealed that the ReHo value alterations in occipital cortex in remitted geriatric depression[53]. Taken together, these findings suggest that the abnormal spontaneous neural activity in the SMA, IOG and MOG might be associated with the pathophysiology mechanism of LOD.

Interestingly, we observed increased ReHo value in the right frontoparietal regions (extend from the middle frontal gyrus to postcentral gyrus) in both patient groups compared to each control group. Previous studies exhibited the ReHo value increased in depression patients when compared with HC[26, 27]. Liang et al. found the ReHo value of unipolar depression patients were increased in lots of brain regions when compare to HC, especially in the right inferior parietal lobule and the right precuneus[26]. A previous study exhibited the blood flow of the back side of the bilateral parietal would increased according with the information complexity but the bilateral parietal blood flow would decline when the subjects were adopting the information[54]. Thus, they speculated the ReHo value increased in parietal region of depression patients might lead to the ability of receiving new information and learning is decreased. Further, the frontoparietal region composes the frontoparietal network (FPN) that is involved in cognitive control of attention and emotion regulation. The FPN has been found to be impaired in major depression[55]. A meta-analysis revealed that MDD was characterized by hypoconnectivity within the FPN, and associated with hyperconnectivity between frontoparietal control systems and regions of the DMN[56]. Moreover, a significantly decreased connectivity in the bilateral frontoparietal regions in MDD compared to HC was also found in a previous study[57], suggesting frontoparietal region might be a key region related to pathomechanism of depression. Meanwhile, the ReHo value was calculated depended on the regional cerebral blood flow (rCBF). Vasic N et al. found that the rCBF increased in the frontoparietal and striatal regions in MDD patients, and the rCBF of the right middle frontal cortex was positively correlated with depressive symptoms[58]. This finding strongly supported our observation of ReHo abnormalities in the right frontoparietal regions. Taken together, these data suggested that (1) MDD might characterized by dysfunction in frontoparietal network; (2) ReHo abnormalities in frontoparietal regions might be the appropriate brain regions to distinguish MDD from HC independent of onset age; (3) patients with MDD might have cognitive control problem or learning deficits associated with the frontoparietal regions independent of age of onset.

In brief, we used the ReHo method to analyze the regional neural activities in a group of first episode and drug-naive patients with MDD. We found different regional neural activity abnormalities between patients with EOD and patients with LOD, suggesting patients with different age-onset might have different pathomechanism. Furthermore, the regional ReHo abnormalities in the right frontoparietal regions might be the appropriate brain regions to distinguish MDD from HC independent of onset age.

There are several limitations of the present study. First, the psychological assessments were not conducted in the present study, therefore, we were unable to examine the relationship between the abnormal ReHo value and psychological assessments. Second, all patients were drug-naive, in their first-episode, and with short disease duration. A longitudinal study is needed to clarify whether the abnormal ReHo is stable after treatment. Third, some statistical maps that did not meet the combined threshold of p<0.05 (False Discovery Rate corrected) with cluster size >100 voxels but met a relaxed threshold of p<0.05 (uncorrected) with cluster size >100 voxels. Though these results may be a little exploratory, but these results would still be valuable to lighten the pathological mechanism between EOD and LOD.

## Supporting information

S1 Checklist Checklist of items that should be included in reports of observational studies. (DOCX)
